# Which contributes more to the relict flora distribution pattern in East Asia, geographical processes or climate change? New evidence from the phylogeography of *Rehderodendron kwangtungense*

**DOI:** 10.1186/s12870-024-05181-7

**Published:** 2024-05-27

**Authors:** Jiehao Jin, Wanyi Zhao, Sufang Chen, Chao Gu, Zhihui Chen, Zhongcheng Liu, Wenbo Liao, Qiang Fan

**Affiliations:** 1 State Key Laboratory of Biocontrol and Guangdong Provincial Key Laboratory of Plant Resources, School of Life Sciences, Sun Yat-sen University, Guangzhou, 510275 China; 2 Shenzhen Dapeng Peninsula National Geopark, Shenzhen, 518121 China

**Keywords:** Population genetics, RAD-seq, Fragmented distributions, Mountain erosion, Panplain, Species diffusing

## Abstract

**Background:**

Relict species are important for enhancing the understanding of modern biogeographic distribution patterns. Although both geological and climatic changes since the Cenozoic have affected the relict flora in East Asia, the contributions of geographical processes remain unclear. In this study, we employed restriction-site associated DNA sequencing (RAD-seq) and shallow genome sequencing data, in conjunction with ecological niche modeling (ENM), to investigate the spatial genetic patterns and population differentiation history of the relict species *Rehderodendron kwangtungense* Chun.

**Results:**

A total of 138 individuals from 16 populations were collected, largely covering the natural distribution of *R. kwangtungense*. The genetic diversity within the *R. kwangtungense* populations was extremely low (*H*_O_ = 0.048 ± 0.019; *H*_E_ = 0.033 ± 0.011). Mantel tests revealed isolation-by-distance pattern (R^2^ = 0.38, *P* < 0.001), and AMOVA analysis showed that the genetic variation of *R. kwangtungense* occurs mainly between populations (86.88%, K = 7). Between 23 and 21 Ma, *R. kwangtungense* underwent a period of rapid differentiation that coincided with the rise of the Himalayas and the establishment of the East Asian monsoon. According to ENM and population demographic history, the suitable area and effective population size of *R. kwangtungense* decreased sharply during the glacial period and expanded after the last glacial maximum (LGM).

**Conclusion:**

Our study shows that the distribution pattern of southern China mountain relict flora may have developed during the panplain stage between the middle Oligocene and the early Miocene. Then, the flora later fragmented under the force of orogenesis, including intermittent uplift during the Cenozoic Himalayan orogeny and the formation of abundant rainfall associated with the East Asian monsoon. The findings emphasized the predominant role of geographical processes in shaping relict plant distribution patterns.

**Supplementary Information:**

The online version contains supplementary material available at 10.1186/s12870-024-05181-7.

## Introduction

Relict species are considered fascinating ‘living fossils’ or remnants of ancient clades and biotas and can be divided into geographical relicts and phylogenetic relicts [1, 2]. These organisms are often viewed as providing compelling evidence for the conservation of ancestral character states in terms of morphology, ecology [3, 4], and spatial distribution [5, 6]. Relict species are often confined to ecological refugia and have fragmented distributions, and they are commonly high priority targets for biodiversity conservation [7]. They must adjust their physiological adaptability [8] and purge the accumulation of deleterious mutations within the population to quickly respond to the pressure of environmental changes [9]. Thus, relict species serve as a “window to the past”, allowing us to understand the conditions that enabled them to survive for so long [1, 10, 11].

East Asia contains the most abundant Tertiary relict flora in the Northern Hemisphere [12–14]. Southeast China is an important host of such relict flora and is considered a floristic museum characterised by phylogenetic and distribution centres of ancient relict species [15, 16]. In general, climatic fluctuations during the Cenozoic prompted the southward migration of Northern Hemisphere flora [17]. High topographic heterogeneity and climate stability has allowed East Asia to serve as a long-term stable habitat, and many species remained in situ during the Pleistocene glaciation [18–22]. Many phylogeographic studies have indicated that plants can survive the climatic fluctuations associated with an ice age and take refuge in mountains in situ [23–26]. It is clear that the establishment of relict flora in East Asia occurred much earlier than the Pleistocene. Previous studies have shown that past geographic events and climate changes have jointly influenced the lineage differentiation of relict plants in East Asia [13, 27–29]. However, the distribution ranges of relict species prior to fragmentation have not been explored, and the independent effects of climate and geography on relict patterns still need further study.

In fact, a topographic reversal has occurred in South China during the Cenozoic era, and the implications on biota of this phenomenon may have been previously overlooked. Before the late Eocene, the terrain of South China was high along the eastern coast of Cathaysia and low in the western Yangtze interior [30–32]. Several lacustrine sedimentary basin, such as the Danxia Basin, Nanxiong Basin, Heyuan Basin, and Guiping Basin, formed in the interior under hot and arid climates [26, 33]. During the early Oligocene, the uplift of the SE Tibetan Plateau triggered a topographic reversal in the South China Block [34–38]. This period was also the key period for the transformation of the palaeo-Pearl River, which flowed from east to west, to the present Pearl River, which flows from west to east [39, 40]. Although, the exact time frame during which the modern Pearl River formed, such as during the early Miocene [41], late Oligocene [39], or ∼ 30 Ma [42], is debated. There is no doubt that a peneplain existed between the Cathaysian coast (current coastal mountains of South China) and the Yangtze interior between the Oligocene and Miocene [43]. This peneplain can still be observed as the present mountain planation surface in the Nanling Mountains, specifically at elevations of approximately 1000–1350 m in northern Guangdong [44, 45] and 1416–1780 m in northern Guangxi and southern Hunan [46]. A large number of relict plants appeared in the high altitudes of the Nanling Mountains, e.g. *Fokienia hodginsii* [22], *Bretschneidera sinensis* [25]. The occurrence of this continuous relict flora is highly improbable to be attributed to random chance. Thus, we suspect that the peneplain stage between the Oligocene and Miocene in South China was important for establishing continuous warm East Asian relict flora.

**Fig. 1:**
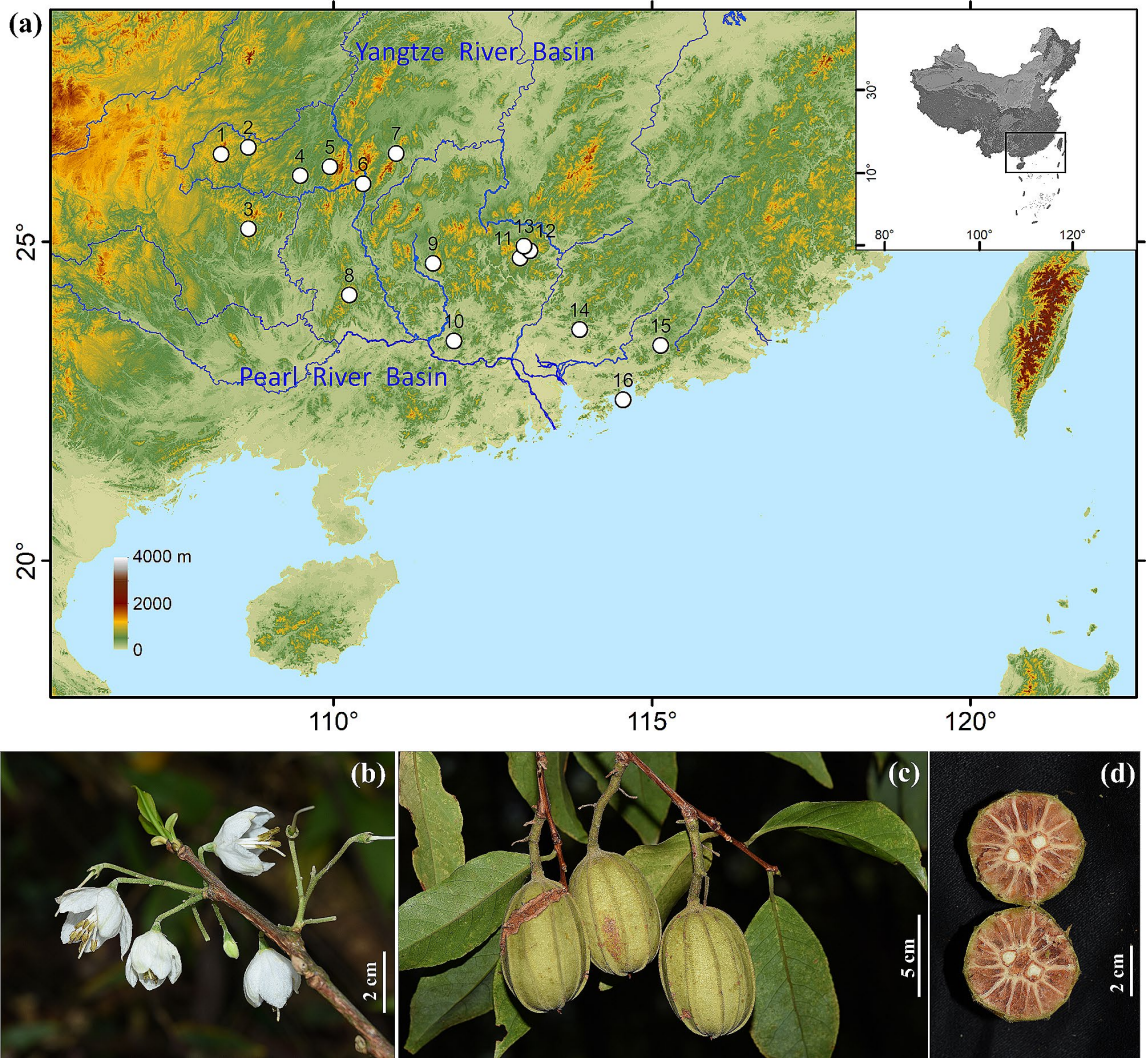
Geographic distribution and morphology of *R. kwangtungense*. (**a**) The white dots indicate the 16 sampling sites in this study. (**b**) Flowering branch. (**c**) Fruiting branch. (**d**) Transverse section of a fruit

*Rehderodendron* Hu (Styracaceae) is a typical relict genus, whose fossil fruits and pollen are widely known in Europe from the early Eocene to the Pliocene [47–51]. However, the modern species of *Rehderodendron* are restricted mostly to warm tropical and subtropical climates in the mountains of East Asia [52]. Our previous phylogenetic evidence indicated that *R. kwangtungense* Chun is the most ancient species within *Rehderodendron* (unpublished data). This fact is further demonstrated by the similarities between modern pollen and pollen discovered in the lower Eocene strata of England [48]. *R. kwangtungense* is sporadically distributed in fog-dependent forests in the subtropical mountains of southern China, exhibiting an island-like (near-mountaintop, mountain planation surface) distribution pattern (Fig. 1a). This island-like distribution pattern is widely observed in East Asian relict species but remains poorly studied. *R. kwangtungense* is an ancient species with low dispersal capacity, making it an ideal species for tracing the spatiotemporal dynamic history of relict flora in East Asia.

Here, we examined the genetic diversity and population divergence of *R. kwangtungense* by employing RAD-seq data [53]. Then, we used ENM to identify the climatic niche of this species under current environmental conditions and to predict its potential distribution during the LGM and mid-Holocene. We combined this information to assess the formation of the modern distribution pattern of *R. kwangtungense*. Our specific objectives were to: (1) whether the distribution patterns of *R. kwangtungense* formed in the panplain stage in the Oligocene, (2) reveal the population structure and population demographic history, and (3) conservation genetics research on *R. kwangtungense*. This study is expected to provide new understanding of East Asian relict flora.

## Methods

### The studied species, sample collection and DNA extraction

*Rehderodendron kwangtungense* is a 5–15 m tall arbor with a diameter up to 30 cm at breast height. Its corolla is white, bell-shaped and fragrant (Fig. 1b), and its fruits are cylindrical ellipsoid in shape (Fig. 1c). The woody exocarp is approximately 1 mm thick, the fibrous corky mesocarp is 8–12 mm thick, and the woody endocarp is hard and radiates into the mesocarp (Fig. 1d). The tillering ability of *R. kwangtungense* is relatively strong. On the one hand, when the trunk of a tree is broken, the base can branch and grow into a larger tree; On the other hand, after the main trunk falls down and touches the soil, it is easy to take root and clone many trees. During field investigation, we found two major distribution areas of *R. kwangtungense* with obvious phenological differences, one on the eastern margin of the Yunnan-Guizhou Plateau (northwestern populations, P1-7; Fig. 1a) and the other in the Nanling and southern mountains (southeastern populations, P8-16; Fig. 1a). In March and April, when the southeastern populations are blooming, the northwestern populations are still in the early bud stage.

Fresh leaves were collected from a total of 138 individuals (16 populations) of *R. kwangtungense*, covering most of its geographic range (Table 1; Fig. 1a). The geographical information of the populations was recorded using a Garmin GPS unit (GPSMAP 62sc, Shanghai). Voucher specimens were deposited at the Herbarium of Sun Yat-sen University (SYS). The fresh leaves were dried and deposited with silica gel in sealed bags. Genomic DNA was isolated using the modified cetyl trimethylammonium bromide (CTAB) method [54].

### RAD-seq data processing

RAD-seq library preparation and Illumina sequencing were performed by Novogene Bioinformatics Technology Co. Ltd. (Tianjin, China). The standard protocol was followed. Stacks was developed to work with RAD-seq for the purpose of building genetic maps and conducting population genomics and phylogeographic analyses. Therefore, we processed the RAD-Seq data using Stacks v2.55 [55]. The program “process_radtags” was used to decomplex reads for each sample, and “denovo_map” was used to determine the optimum values for M (number of mismatches allowed between stacks within individuals) and *n* (number of mismatches allowed between stacks among individuals) with subset data containing 12 samples. Then, “denovo_map” was reused to process all 138 samples with the optimum M and *n* values (M = *n* = 4). Finally, the results were filtered using the program “populations”, in which polymorphic RAD loci that were present in at least 40% of the individuals across populations, had a minor allele frequency > 0.05 and had an observed heterozygosity < 0.7 were retained (-R 0.4 --min-maf 0.05 --max-obs-het 0.7). And for each locus, unlinked SNP were retained using the command “--write-single-snp”. To detect non-neutral loci under selection, Tajima’s *D* was calculated using VCFtools [56], in which the window size was set as 3,000 bp. Loci with Tajima’s *D* value < − 1.771 or > 2.086 were deemed as non-neutral [57]. The produced vcf files (Supplementary Material S1) were used for downstream analyses, and converted to other formats using VCFtools.

### Genetic diversity and population structure analysis

The program “populations” of Stacks was used to calculate genetic diversity parameters (*H*_O_, *H*_E_, π and *F*_IS_) and pairwise fixation index (*F*_ST_) [55]. Population structure was examined using Admixture v1.3.0 [58]. The value of the hypothetical ancestral population (K) was set from 1 to 10, the optimal K was determined by the smallest cross-validation (CV) error value, and Q estimates were used as a proxy for ancestry fractions. Principal component analysis (PCA) was performed using Plink v1.90b to evaluate population genetic variation [59]. Analysis of molecular variance (AMOVA) was performed to estimate standardized genetic differentiation using Arlequin v3.5 [60], in which the 16 populations of *R. kwangtungense* were divided into two regions (northwestern and southeastern regions according to variation in flowering time), four regions according to PCA, and seven regions according to Admixture analysis. The python script “vcf2phylip.py” (https://github.com/edgardomortiz/vcf2phylip) was used to convert the vcf file to the phylip format, and the maximum likelihood phylogenetic tree was produced with IQ-TREE v2.2 [61] and plotted with iTOL v6 [62].

### Population historical dynamics inference

The site frequency spectra (SFS) were generated using easySFS (https://github.com/isaacovercast/easySFS), and the value for projecting down samples was set to 112 to maximize the number of segregating sites. Stairway Plot v2.1.1 [63] was subsequently used to infer demographic history based on the folded SFS. Stairway Plot is based on SFS, which does not require whole-genome sequence data or a reference genome to infer recent population size changes [64]. The mutation rate of *R. kwangtungense* was set to 2.29 e-9 per site per year according to the estimate for walnut by Luo et al. [65]. The generation time was set to ten years, which was observed in the field investigation.

### Shallow genome sequencing and plastid genome, ribosomal cistron assembly

For each population, we randomly selected one individual for shallow genome sequencing. In addition, 5 other species of *Rehderodendron* have undergone shallow sequencing and were utilized in the construction of the phylogenetic tree. The extracted DNA was sent to JieRui BioScience Co. Ltd. (Guangzhou, China) for library construction and Illumina sequencing on the Illumina 2000 platform. The program Novoplasty v2.7.2 [66] was used to assemble the chloroplast genome, using the *R. macrocarpum* chloroplast genome (GenBank accession MG719844) as a reference and its chloroplast *rbcl* gene sequence as a seed. Meanwhile, GetOrganellle v1.7.7 [67] was used to assemble the ribosomal cistron, using the *Melliodendron xylocarpum* ribosomal cistron (GenBank accession MF171073) as a seed.

### Phylogenetic tree construction and divergence time estimation

According to Yan et al. [68], the plastid genomes of 6 related genera were downloaded from the NCBI nucleotide database (Supplementary Table 1). Together with the 16 newly assembled plastid genomes of *R. kwangtungense* and 5 other species of *Rehderodendron* in this study, 31 plastid genome sequences were aligned using MAFFT v7.508 [69]. Gaps and ambiguous bases (N) were removed using MEGA 7 [70]. A similar method was used to standardize ribosomal DNA data (Supplementary Table 2). The processed sequences were subsequently used to construct a phylogenetic tree, and the divergence times were inferred via Beast v1.8.4 [71]. The tree was calibrated with three fossils, *Rehderodendron stonei* (52 − 49 Ma) [49], *Pterostyrax coronatus* (33.9–28.1 Ma) [72] and *Halesia reticulata* (37.2–33.9 Ma) [73]. The lognormal prior distributions were enforced with a mean = 1.5, a Stdev = 2.0 and an offset set so that 95% of the distribution would fit the age of the upper (youngest) stratum from which each fossil was described. A GTR + I + F + G4 substitution model and lognormal relaxed-clock model were used to allow rate variation among branches. Additionally, a constant size was chosen as the tree prior. The analysis was run for 400 million Markov Chain Monte Carlo (MCMC) steps. MCMC samples were imported into TRACER v1.5 (available from http://beast.bio.ed.ac.uk/Tracer) to inspect the sampling adequacy and convergence of the chains to a stationary distribution. The final plot was visualized using Figtree v1.4.4.

### Ecological niche modelling

The ecological niche model (ENM) was used to predict the potential distribution of *R. kwangtungense*. In addition to our sample locations, geographic distributions of the species were also obtained from the Chinese virtual herbarium portal (CVH, https://www.cvh.ac.cn/). After filtering duplicate records and invalid records without a specific location, a total of 51 records were used in the following step. Climate layer information for 19 bioclimatic variables was downloaded from WorldClim [74, 75] at a resolution of 2.5 arc-minutes. Based on geographical distribution data from *R. kwangtungense* and 19 climatic factors, a MaxEnt model was initially established to evaluate the contributions of different variables to the distribution of climate data [76]. To avoid overfitting of the simulation results due to the mutual influence between highly correlated climatic variables, Pearson correlation coefficient analysis was performed among the 19 climatic variables using SPSS v26.0. A pair of climatic variables with correlation coefficients greater than |0.8| was considered non-independent. Finally, for each pair of significantly correlated variables, only one variable with a large contribution was used for projection. After the selection procedure, 7 variables were retained for modelling the distribution of *R. kwangtungense*: the mean diurnal range (Bio2), isothermality (Bio3), minimum temperature of coldest month (Bio6), temperature annual range (Bio7), annual precipitation (Bio12), precipitation of driest quarter (Bio17), and precipitation of coldest quarter (Bio19). We employed MaxEnt 3.4.4 to simulate and predict the evolution of potentially suitable areas of *R. kwangtungense* in different periods, using default settings and specific parameters: random test (25%), training (75%), regularization multiplier (1), maximum iterations (5000), convergence threshold (0.00001), maximum background points (10,000), and 10 bootstrap replications. Model accuracy was gauged using the AUC of receiver operating characteristic (ROC). The output file was imported into ArcGIS 10.8, and the suitability was manually divided at 0.05 intervals, and areas with suitability values less than 0.1 were considered unsuitable areas.

### Correlation of genetic structure with geographic isolation and environment factor

The isolation-by‐distance (IBD) reveals that genetic differentiation among populations increases with geographic distance, while the isolation‐by‐environment (IBE) assumes a linear relationship between genetic variation and environmental differences among populations [77]. To explore the existence of IBD and IBE, we applied Mantel test to assess the relationships between genetic distance and geographic or environmental distance with R v4.3.2 (https://www.r-project.org/). The pairwise genetic distance among populations was calculated by *F*_ST_/(1 − *F*_ST_), the geographic distance matrix was transformed by the geographic coordinates of populations, and 7 climatic variables used in ENM were matrix transformed using PCA to obtain the environmental distance. A partial mantel test was conducted to control the influence of geographical and environmental distance, respectively.

## Results

### RAD data processing and genetic diversity

A total of 11,190,163 loci were genotyped by the “denovo_map” program, and the mean, minimum and maximum values of effective per-sample coverage were 17.1×, 10.1× and 23.7×, respectively. After data filtration, a total of 1,943 SNPs remained for all subsequent analyses. The Tajima’s D values of all 1943 variant sites ranged from − 0.394 to 2.029, all sites are neutral. The observed heterozygosity (*H*_O_) for the 16 populations ranged from 0.022 to 0.078, with an average value of 0.048 ± 0.019; the expected heterozygosity (*H*_E_) ranged from 0.019 to 0.052, with an average value of 0.033 ± 0.011; and the inbreeding coefficient (*F*_IS_) was close to 0 (-0.0009 to 0.0005) (Table 1).

**Table 1:** Geographic information and genetic diversity parameters of *R. kwangtungense*

Code	Pop	Location	Latitude(*N*)	Longitude(E)	Elev(m)	*N*	H_O_	H_E_	π	F_IS_
P1	LGS	Leishan, Guizhou	26°22′27″	108°13′58″	1718	12	0.0227	0.0195	0.0217	-0.0009
P2	SC	Liping, Guizhou	26°29′05″	108°39′31″	1473	11	0.0223	0.0185	0.0210	-0.0019
P3	JWS	Rongshui, Guangxi	25°12′17″	108°39′52″	1505	12	0.0238	0.0206	0.0231	-0.0012
P4	SSP	Tongdao, Hunan	26°02′22″	109°28′29″	1181	10	0.0538	0.0377	0.0412	-0.0236
P5	SD	Tongdao, Hunan	26°10′45″	109°56′29″	1128	4	0.0706	0.0428	0.0602	-0.0176
P6	MES	Xing’an, Guangxi	25°54′43″	110°27′45″	1606	8	0.0495	0.0355	0.0401	-0.0183
P7	SHS	Xinning, Hunan	26°23′14″	110°58′55″	1435	12	0.0335	0.0295	0.0326	0.0005
P8	DYS	Jinxiu, Guangxi	24°10′01″	110°14′41″	1186	11	0.0646	0.0462	0.0522	-0.0210
P9	GPS	Jianghua, Hunan	24°39′50″	111°33′44″	1048	10	0.0601	0.0386	0.0440	-0.0296
P10	HSD	Fengkai, Guangdong	23°26′45″	111°53′29″	632	5	0.0782	0.0528	0.0700	-0.0130
P11	TJS	Yangshan, Guangdong	24°44′54″	112°55′36″	1270	9	0.0681	0.0434	0.0501	-0.0327
P12	NL	Yangshan, Guangdong	24°51′46″	113°5′3″	1100	10	0.0597	0.0403	0.0469	-0.0235
P13	MS	Yizhang, Hunan	24°56′9″	112°59′19″	1596	4	0.0613	0.0369	0.0519	-0.0149
P14	NKS	Longmen, Guangdong	23°37′32″	113°51′48″	584	8	0.0291	0.0201	0.0248	-0.0069
P15	WQZ	Zijin, Guangdong	23°22′31″	115°08′06″	600	4	0.0352	0.0215	0.0308	-0.0068
P16	QNS	Shenzhen, Guangdong	22°31′20″	114°32′44″	725	8	0.0294	0.0211	0.0256	-0.0060

Notes: Pop, population ID; Elev, elevation; *N*, number of sampled individuals; *H*_O_, observed heterozygosity; *H*_E_, expected heterozygosity; π, nucleotide diversity; *F*_IS_, inbreeding coefficient

### Population structure

**Fig. 2:**
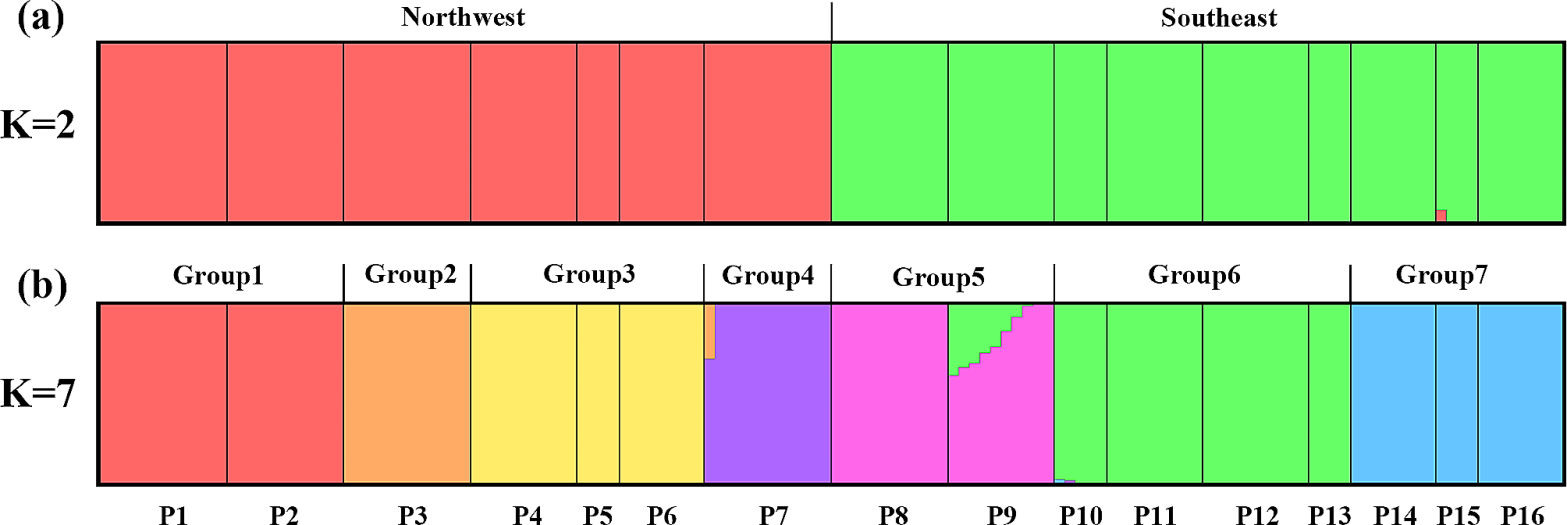
Delimitation of 16 populations of *R. kwangtungense* based on Admixture analysis. (**a**) Genetic structure of *R. kwangtungense* based on *K* = 2. (**b**) Genetic structure of *R. kwangtungense* based on *K* = 7. Each individual (indicated as columns along the X-axis) is probabilistically assigned (probability of assignment q on the Y-axis) to one of the inferred genetic clusters

Based on the results of the Admixture program, the CV error decreases and becomes stable at K = 7 (Supplementary Fig. 1). When K = 2, the populations of *R. kwangtungense* were divided into northwestern group (P1-7) and southeastern group (P8-16) (Fig. 2a), which was consistent with phenological differences. When K = 7, the populations were subdivided gradually, with the northwestern cluster divided into four groups (G1-4) and the southeastern cluster divided into three groups (G5-7) (Fig. 2b).

**Fig. 3:**
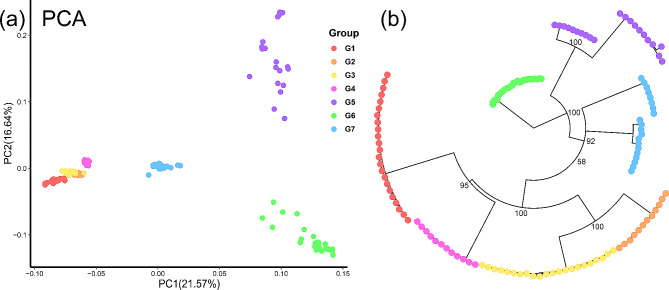
Genetic and phylogenetic analysis of *R. kwangtungense*. (**a**) Two-dimensional clustering of individuals obtained via principal component analysis. (**b**) Phylogenetic tree constructed from RAD-seq data

PCA showed that the contribution rates of the first and second principal components were 21.57% and 16.64%, respectively. The two-dimensional plot of PC1 and PC2 divided all the 138 individuals into four large clusters (Fig. 3a): the seven populations located in the northwestern area were ascribed to one cluster, including the four groups G1-G4 according to Admixture analysis (Fig. 2b), while the nine populations in the southeastern area were divided into three clusters, corresponding to the three groups G5-G7 according to Admixture analysis (Fig. 2b). The unrooted phylogenetic tree based on population data also confirmed that individuals of most group (G1-G6) clustered into a monophyletic group with a high support value (Fig. 3b). All terminal branches are very short in length (Fig. 3b), indicating little genetic variation within the population.

AMOVA showed that the genetic variation among regions increased from 27.41 to 59.72% and that the variation among populations within groups decreased from 60.41 to 27.16% as the K value increased from 2 to 7, and the variation within populations remained stable (12.18-13.12%, Table 2).

**Table 2:** Analysis of molecular variance (AMOVA) of *R. kwangtungense*

Source of variation	Percentage of variation		
	K = 2	K = 4	K = 7
Among groups	27.41	44.69	59.72
Among populations within groups	60.41	43.15	27.16
Within populations	12.18	12.16	13.12

### IBD and IBE

**Fig. 4:**
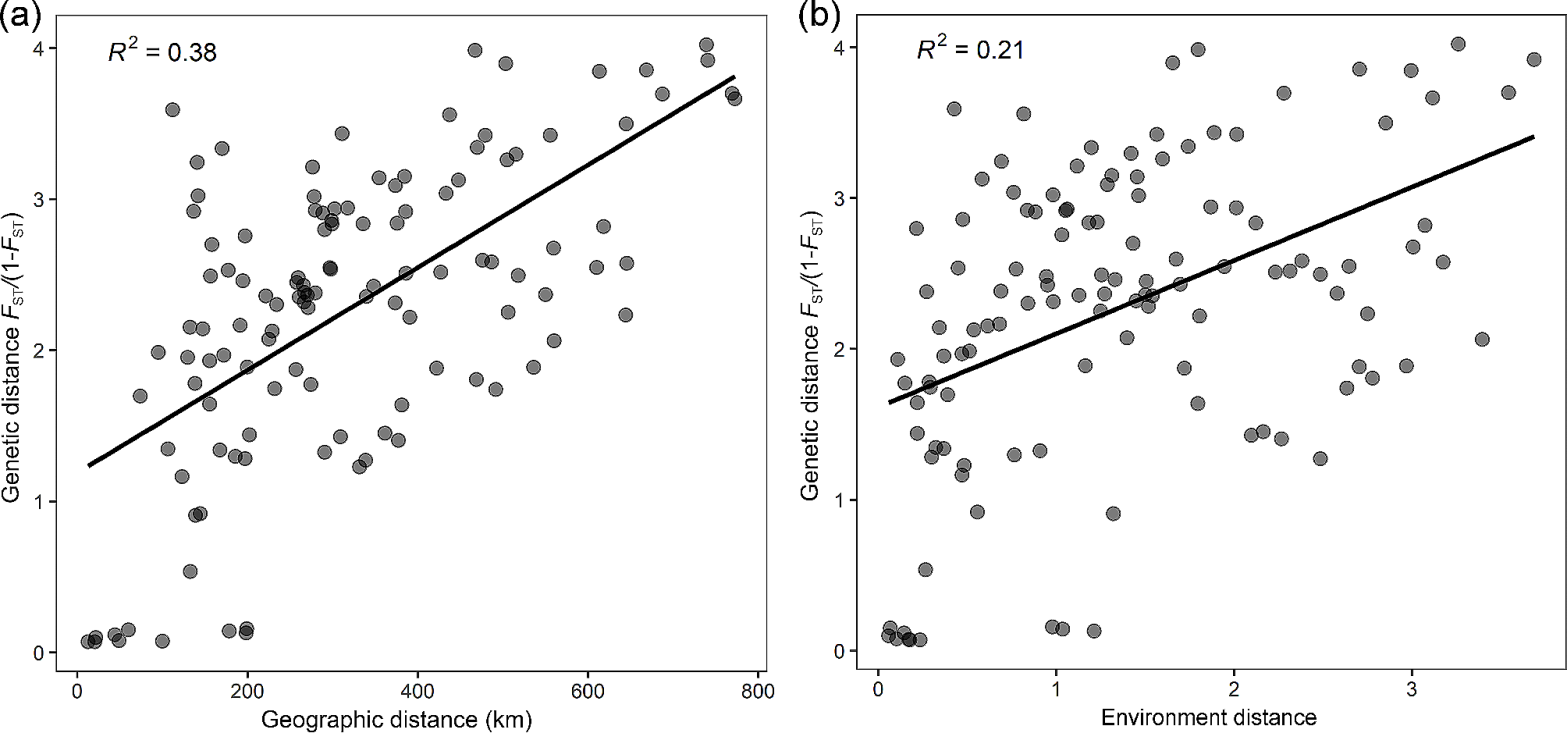
Isolation-by-distance (IBD) and Isolation-by-environment (IBE) patterns for populations of *Rehderodendron kwangtungense*. (**a**) Mantel test result of genetic distance and geographical. (**b**) Mantel test result of genetic distance and environment distance. Each dot represents a pair of populations

The mantel test showed that genetic distance [*F*_ST_/(1–*F*_ST_)] was significantly correlated with geographical distance and conformed to an isolation-by-distance pattern (R^2^ = 0.38, *P* < 0.001, Fig. 4a). Meanwhile, environmental factors also affected the genetic variation (R^2^ = 0.21, *P* < 0.001, Fig. 4b). The partial mantel test showed that after controlling the influence of environment matrix, geographical distance and genetic distance were still related (R^2^ = 0.24, *P* < 0.001). However, after controlling for the influence of geographical matrix, there was no correlation between environmental factors and genetic distance (R^2^ = 0.018, *P* = 0.881). Therefore, geographical isolation rather than environmental factors was the main factor that caused the genetic differences of *R. kwangtungense* populations.

### Population demographic history

**Fig. 5:**
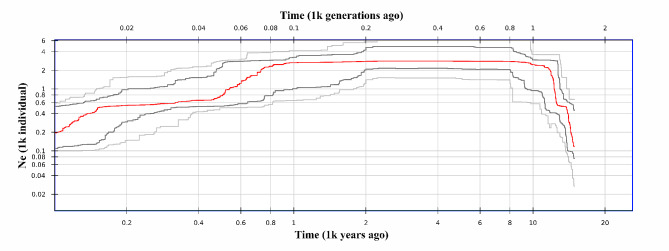
Estimates of the effective population size of *R. kwangtungense*

A demographic history investigation showed that the effective population size of *R. kwangtungense* increased quickly from approximately 16,000 years ago (YA) to 10,000 YA and then remained stable above 2000 until 8,00 YA. It subsequently gradually declined to the very low level observed at present (approximately 200) (Fig. 5).

### Divergence history of populations inferred by phylogenetic tree

**Fig. 6:**
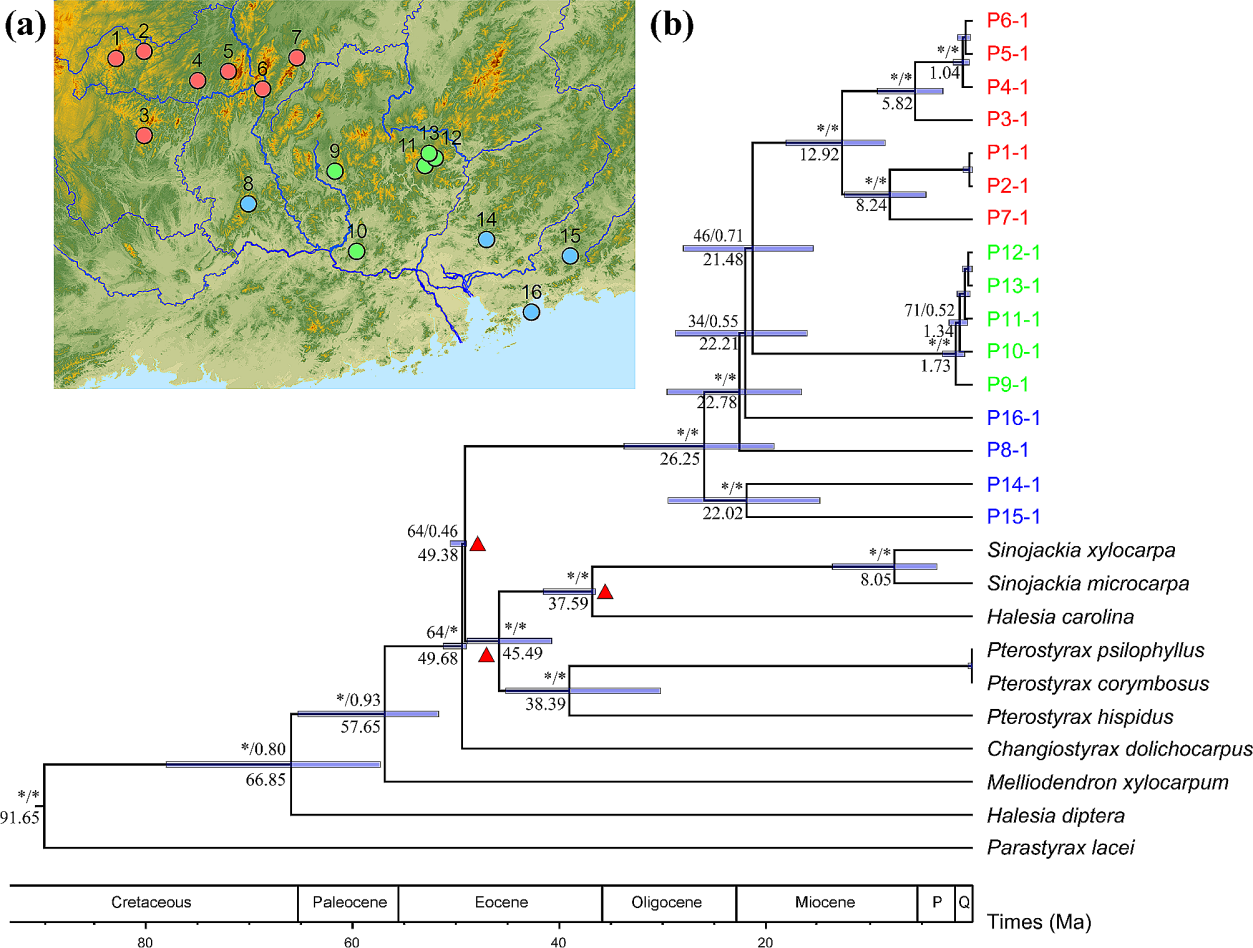
(**a**) The presumed migration path of *R. kwangtungense*. (**b**) Phylogenetic tree and divergent time estimation (below the bars) of *R. kwangtungense* based on the plastid genome. The fossil points are marked with triangles. Maximum likelihood standard bootstrap support values (BS) and Bayesian posterior probabilities (PP) are shown above the bars (*: BS ≥ 95% or PP ≥ 0.99). The median ages of the nodes are shown below the branches (those younger than 1 Ma are not displayed), with blue bars indicating the 95% highest posterior density intervals

The plastid and ribosomal cistron phylogenetic tree yielded similar topologies and intra-genus divergence time. However, ribosomal phylogenetic tree showed lower posterior probability and larger time estimation range (Supplementary Fig. 2), and ribosomal DNA has limited credibility as one of the nuclear genes. Thus, we mainly referred to the plastid phylogenetic tree. Furthermore, the inclusion of other species within *Rehderodendron* genus exerts minimal impact on the estimation of populations divergence time, given that *R. kwangtungense* represents the most ancestral clade (Supplementary Fig. 3). Therefore, only the phylogenetic tree constructed from plastid genomes excluding other *Rehderodendron* species was presented (Fig. 6a and b), which showed that existing *R. kwangtungense* populations differentiated at ca. 26.25 Ma. In addition, populations P14, P15, P16 and P8 were placed on the root of the tree; these populations diverged from the remaining populations at ca. 22–23 Ma. The populations P1-P7 formed a monophyletic clade, and the crown age was estimated to be ca. 12.92 Ma. Additionally, populations P9-P13 formed another monophyletic clade that diverged only recently (ca. 1.73 Ma).

### Suitable distribution based on ENM

**Fig. 7:**
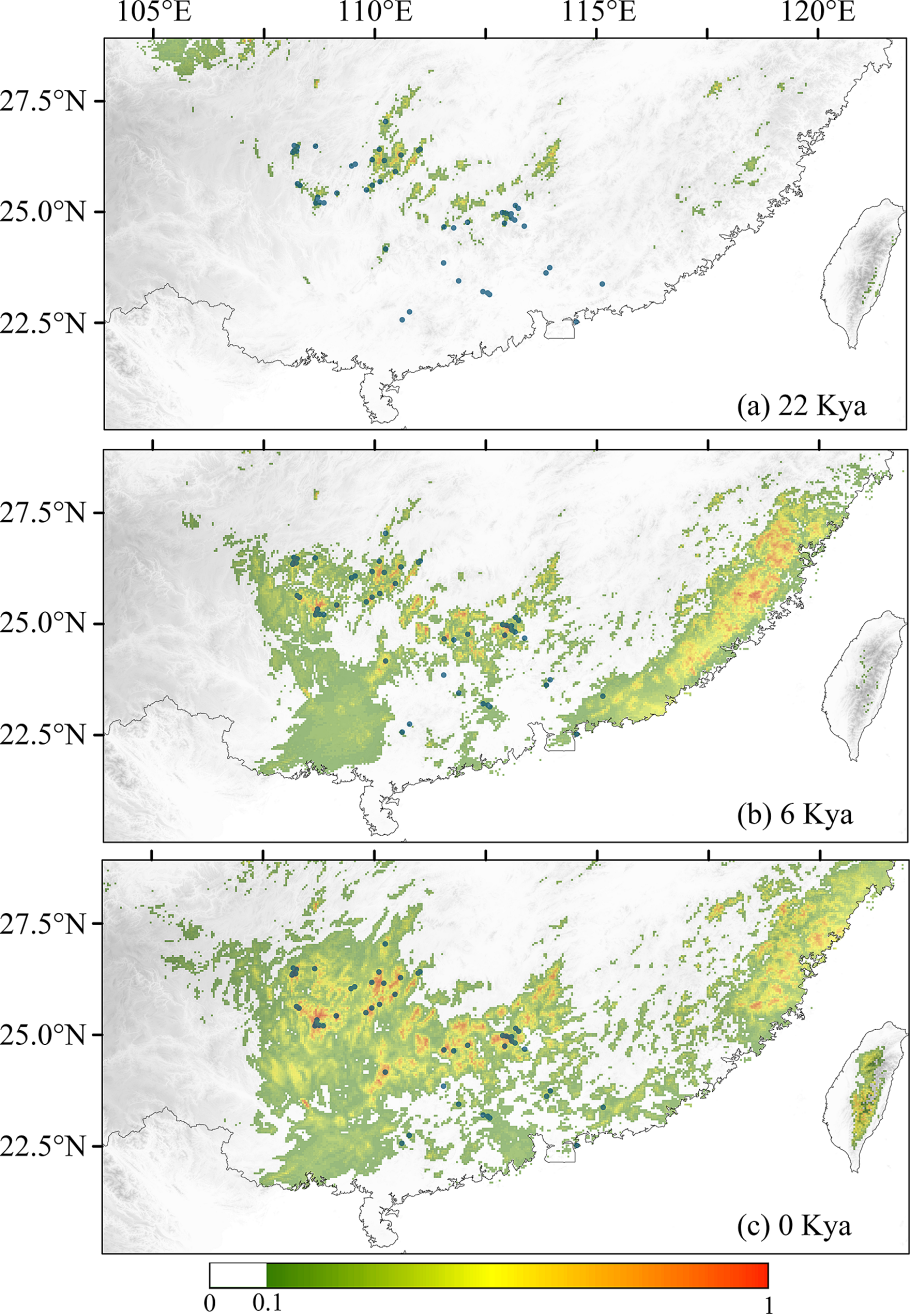
Presence records (dark circles) and predicted distribution probability according to climatic conditions during the (**a**) last glacial maximum (22 kya), (**b**) mid-Holocene (6 kya), and (**c**) current (0 kya)

Among the 7 bioclimatic variables selected for ENM simulation, the bioclimatic variables with greater contribution percent are Bio17, Bio7, Bio6; the bioclimatic variables with greater permutation importance are Bio7, Bio2, Bio6; and the bioclimatic variables with greater regularized training gain of the jackknife test are Bio2, Bio17, Bio19 (Supplementary Tables 3 & Supplementary Fig. 4). In conclusion, the bioclimatic variables that dominate the potential geographical distribution of *R. kwangtungense* are mean diurnal range (bio2), min temperature of coldest month (Bio6), temperature annual range (Bio7) and precipitation of driest quarter (Bio17). The suitable areas for *R. kwangtungense* were few and scattered in southern China during the LGM (5.01 km^2^, Fig. 7a), increased obviously at ca. 6000 YA (26.10 km^2^, Fig. 7b), and reached a maximum at present (43.51 km^2^, Fig. 7c). The predicted areas at ca. 6000 YA and at present are larger than the actual distribution area of the species, mostly in southeastern China.

## Discussion

### Origin and early dispersal of *R. kwangtungense*

Although there have been some phylogenetic studies of family Styracaceae, our study is the first to infer the internal divergence time of family Styracaceae, so there is no intra-family divergence time reference. Based on large-scale phylogenetic tree, Rose et al. [78] showed that the divergence time between the family Styracaceae and the family Diapensiaceae was about 93.2 Ma, and Li et al. [79] showed that the formation time of Ericales was between 103 and 107 Ma, which is close to our estimated time. *Rehderodendron* is suggested to be a member of “boreotropical flora” [48] because of the existence of a continuous zone of maritime influenced vegetation along the Tethys during the Cenozoic [80]. Our phylogenetic results showed that populations P14 and P15, which are located in the coastal mountains of Southeast China, harbor the most ancient plastid genomes (Fig. 6b). These findings suggest that the ancestor species of *R. kwangtungense* may have initially spread to the Cathaysian coast through the lowland region along the Tethys Ocean during the early Eocene, when the Tibetan Plateau had not yet fully uplifted [34]. This early diffusion event is supported by the similarity of *R. kwangtungense* pollen and pollen from the early Eocene [48].

Most of the eastern populations (P8, P14, P15, P16) and early clades (P1-P7, P9-P11) of *R. kwangtungense* quickly diverged between 26.25 Ma and 21.48 Ma (Fig. 6b). This result implies that the modern distribution area of *R. kwangtungense* was already established before the early Miocene.

Field observation revealed that the fruits of *R. kwangtungense* are large and cylindrical with thick spongy mesocarps (Fig. 1c-d), and long-distance transport by animals is difficult (only rodents eat them). Thus, water and gravity are the main forces driving lateral fruit distribution [48]. The Mantel test also confirmed the weak dispersal ability of *R. kwangtungense* (Fig. 4). Therefore, the fruits of *R. kwangtungense* do not have the ability to spread between mountains in different river basins. The current island-like distribution pattern most likely formed before mountain uplift and erosion. Palynological evidence also suggests that the genus *Rehderodendron* seems to be more adapted to braided river system transmission [80]. The existence of a panplain stage between the Oligocene and Miocene with many nondirectional water systems [39], seems to be the most plausible explanation for the early dispersal of *R. kwangtungense*.

### Differentiation of *R. kwangtungense* since the establishment of the east Asian monsoon

The results of population structure analysis suggest that *R. kwangtungense* populations should be divided into seven groups (Fig. 2). Group 1–4 are located on the eastern side of the Yunnan-Guizhou Plateau on the second level of the terrain ladder in China, and the flowering period of these populations is late. Group 5–7 are located in the Nanling Mountains and the southern region on the third level of the terrain ladder, and the flowering period of these populations is early. The phylogenetic tree shows that the crown age of extant *R. kwangtungense* was estimated to be 26.25 Ma, and this species underwent a period of rapid populations divergence at 23 − 21 Ma (Fig. 6b), coinciding with the rapid rise of the Himalayas and subsequent establishment of the East Asian monsoon. Ding et al. proposed that the Himalayas experienced a rapid increase in elevation to 4000 m from 23 to 19 Ma [81], while the Yunnan-Guizhou Plateau was uplifted at a much greater rate than the Nanling Mountains, thus forming the three-step ladder of China’s topography and restructuring the monsoon circulation system. Sun & Wang also suggested that the topographic inversion that occurred at ca. 25 Ma, resulted in the formation of the East Asian monsoon, which had a large impact on the dry and wet patterns in China [31]. The geographical isolation caused by geological movements hindered gene flow, and monsoonal rains intensified mountain erosion, which together promoted the differentiation of *R. kwangtungense* into two groups from 26 − 21 Ma. Moreover, a differentiated population structure between the Yunnan-Guizhou Plateau and South China is common among relict species, such as *Bretschneidera sinensis* [25], *Eomecon chionantha* [82] and *Eurycorymbus cavaleriei* [24].

Subsequently, continuous geographical processes have led to the formation of more complex mountain systems, and several phases of intensification in the Asian monsoon have led to climate change [83]. The *R. kwangtungense* in the Yunnan-Guizhou Plateau has been continuously differentiated since 12.69 Ma. Similar divergence processes were also observed in *Cyclocarya paliurus* and *Eriobotrya* during this period [84, 85].

### Effects of Quaternary glaciation on the *R. kwangtungense* and implications for conservation

Southern China, which has numerous mountains and valleys, provides long-term stable habitats for many species, allowing them to maintain in situ, and harbours a great abundance of Tertiary relict flora [18, 19]. Under the influence of repeated ice ages in the Quaternary, the population of *R. kwangtungense* contracted dramatically and took refuge in mountainous areas. The geographic isolation caused by population contraction restricted gene flow, which combined with a changing climate, facilitated the second phase of rapid divergence in P1-2, P4-6 and P9-13. Similarly, the lineage differentiation has also occurred in *Cercdiphyllum japonicum*, *Kalopanax septemlobus* and other species [86, 87].

Based on ENM and historical dynamic analysis, when the LGM occurred, the area suitable for *R. kwangtungense* decreased sharply, and the effective population size reached an extremely low level, indicating that *R. kwangtungense* was not adapted to the cold climate and that a large number of populations died out, with only a small minority taking refuge in the mountains. As the climate became warm and humid after the LGM, the suitable habitat area for *R. kwangtungense* continued to expand, and the historical dynamic simulation confirmed that the population size increased simultaneously, reaching a peak at approximately 10,000 YA (Figs. 5 and 7). However, since the modern landscape had been basically formed at this point, the dispersal of *R. kwangtungense* was limited, and the species only expanded in situ, forming an island-like distribution pattern. AMOVA indicated that genetic variation within populations was minimal (Table 2), as was that of *H*_E_ and *H*_O_ in various groups (Table 1), and the phylogenetic tree constructed from RAD data showed a very short branch length for all individuals (Fig. 3b). This evidence points to the limited genetic diversity of *R. kwangtungense* populations, and a population may be the result of the cloning of a few individuals. Thus, we surmised that the dispersal type of fruits and pollination mode determined whether population expansion occurred after the ice age. Similarly, *Cathaya argyrophylla* did not experience long-distance dispersal or population expansion after the glacial period [23]. In contrast, *Cyclocarya paliurus* with samara experienced substantial spatial expansion [85].

As temperatures and precipitation become more favorable, the effective population size has shrunk over the past 1,000 years. The main reason for the inconsistency may be the weak diffusion ability and competition ability of *R. kwangtungense*. According to the field observation, the genus *Rehderodendron* can quickly grow many small trees after the forest gap period, while in the original forest with good vegetation, the seedlings are basically invisible (because the fruit will soon become mildew if it is not dried in time). In other words, we assume that the suitable habitat for *R. kwangtungense* is the plain, with rivers and flat land, and the current mountain habitat is not suitable for the diffusion and competition of *R. kwangtungense.* The ENM result is mainly based on temperature and precipitation, but in reality, temperature and precipitation are not the only limiting factors.

Another reason for the reduction in effective population size could be human activities. To our knowledge, many populations are currently located in natural reserves where the species may be well protected (P8, P10 and P13), although in some regions, such as P5, the species are susceptible to the impacts of nearby villages. The fragile genetic diversity of the populations suggests that all populations should be protected, and priority should be given to representative populations in each group. Field observations have shown that seedlings are rare, possibly due to failure to compete with other species, while manual cultivation has shown that successful germination of seeds can be as high as 80% [88]. Therefore, artificial breeding and other measures can be adopted for protection.

## Conclusion

The understanding of the origin of the contemporary distribution pattern of relict plants is crucial for the scientific conservation of rare and endangered plant species. In this study, we revealed the population differentiation history of the relict species *Rehderodendron kwangtungense* Chun during the Cenozoic. The modern distribution area of *R. kwangtungense* was already established during the Oligocene-Miocene transition period, and subsequently underwent fragmentation in Miocene as a result of allometric plate uplift and mountain erosion. During the Quaternary glacial period, *R. kwangtungense* experienced a significant population decline and sought refuge in situ. The findings of our study emphasize the predominant role of geographical processes in shaping relict plant distribution patterns, while the impact of climate fluctuations primarily manifests through their influence on local population size. This means that scenario of population expansion and contraction under climate oscillations may be overestimated, especially for those species with low dispersal capacity. Furthermore, we propose a perspective on the existence of a panplain in south China during the Oligocene to Miocene epochs, which has significant implications for understanding the formation process of relict flora in East Asia.

### Electronic supplementary material

Below is the link to the electronic supplementary material.

Supplementary Material 1

Supplementary Material 2

Supplementary Material 3

Supplementary Material 4

Supplementary Material 5

Supplementary Material 6

Supplementary Material 7

Supplementary Material 8

## Data Availability

The RAD-seq data has been uploaded to NCBI (https://www.ncbi.nlm.nih.gov/) Sequence Read Archive under the BioProject PRJNA1056300. The assembled chloroplast genomes data has been uploaded to NCBI GenBank under the accessions PP048903-PP048918, PP761266-PP761270. And the ribosomal DNA data has been uploaded to NCBI GenBank under the accessions PP765768-PP765788.

## Abbreviations

RAD-seq: Restriction-site associated DNA sequencing
ENM: Ecological niche modelling
LGM: Last glacial maximum
*H*_O_: Observed heterozygosity
*H*_E_: Expected heterozygosity
π: Nucleotide diversity
*F*_IS_: Inbreeding coefficient
*F*_ST_: Fixation index
K: Hypothetical ancestral population
CV: Cross-validation
PCA: Principal component analysis
AMOVA: Analysis of molecular variance
SFS: Site frequency spectra
IBD: Isolation-by-distance
IBE: Isolation-by-environment
YA: Years ago
