# Supplementary material for: Which contributes more to the relict flora distribution pattern in East Asia, geographical processes or climate change? New evidence from the phylogeography of Rehderodendron kwangtungense
Source: BMC Plant Biol. 2024 May 27;24:459. doi: 10.1186/s12870-024-05181-7 (PMC11129394; doi:10.1186/s12870-024-05181-7)
Supplement: Supplementary file 2 — Supplementary Material 2 [file 12870_2024_5181_MOESM2_ESM.docx]

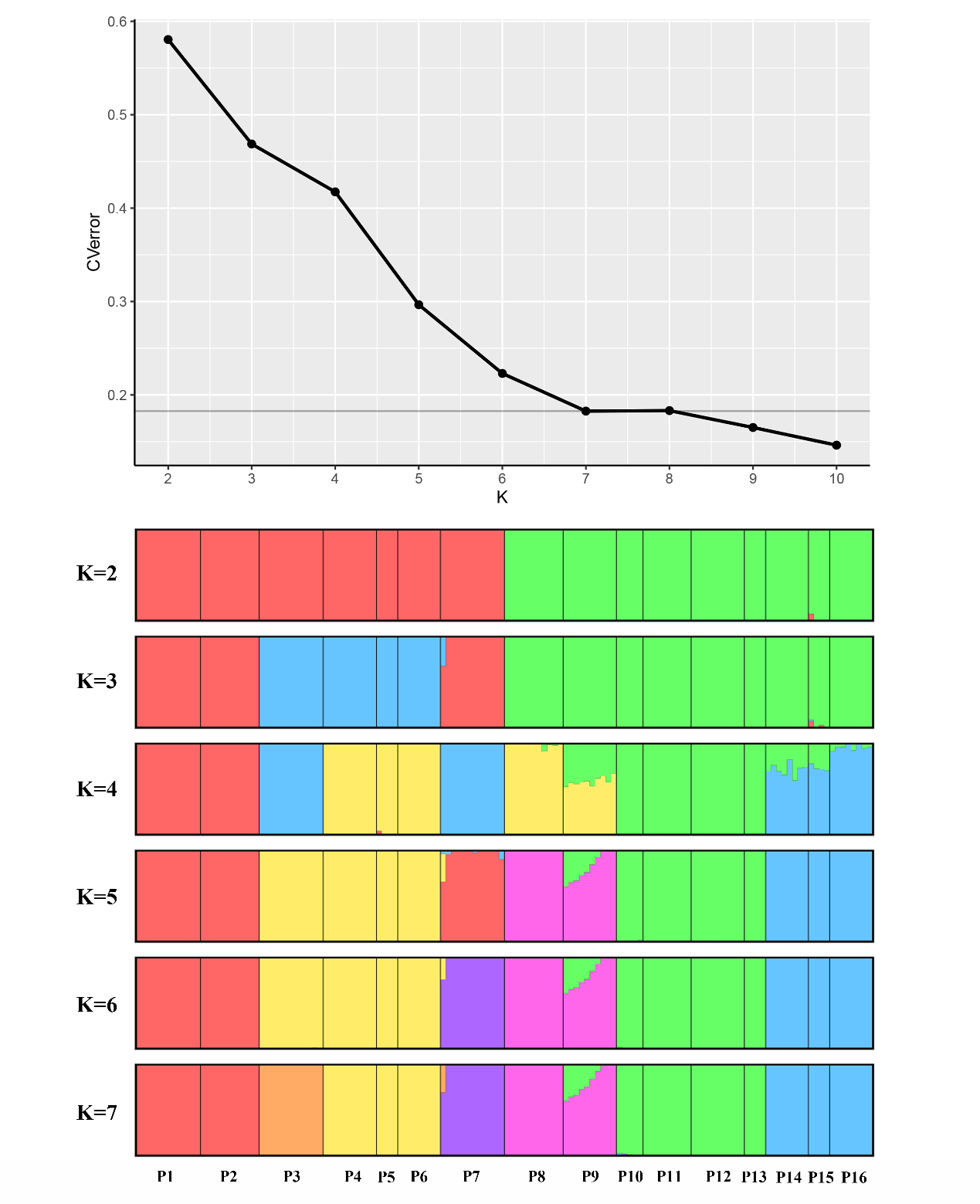


Supplementary Fig. 1 A range of K (K in 2-7) clusters of populations delimitation based on Admixture analysis. Each individual (indicated as columns along the X-axis) is probabilistically assigned (probability of assignment q on the Y-axis) to one of the inferred genetic clusters.
