# Supplementary material for: Which contributes more to the relict flora distribution pattern in East Asia, geographical processes or climate change? New evidence from the phylogeography of Rehderodendron kwangtungense
Source: BMC Plant Biol. 2024 May 27;24:459. doi: 10.1186/s12870-024-05181-7 (PMC11129394; doi:10.1186/s12870-024-05181-7)
Supplement: Supplementary file 3 — Supplementary Material 3 [file 12870_2024_5181_MOESM3_ESM.docx]

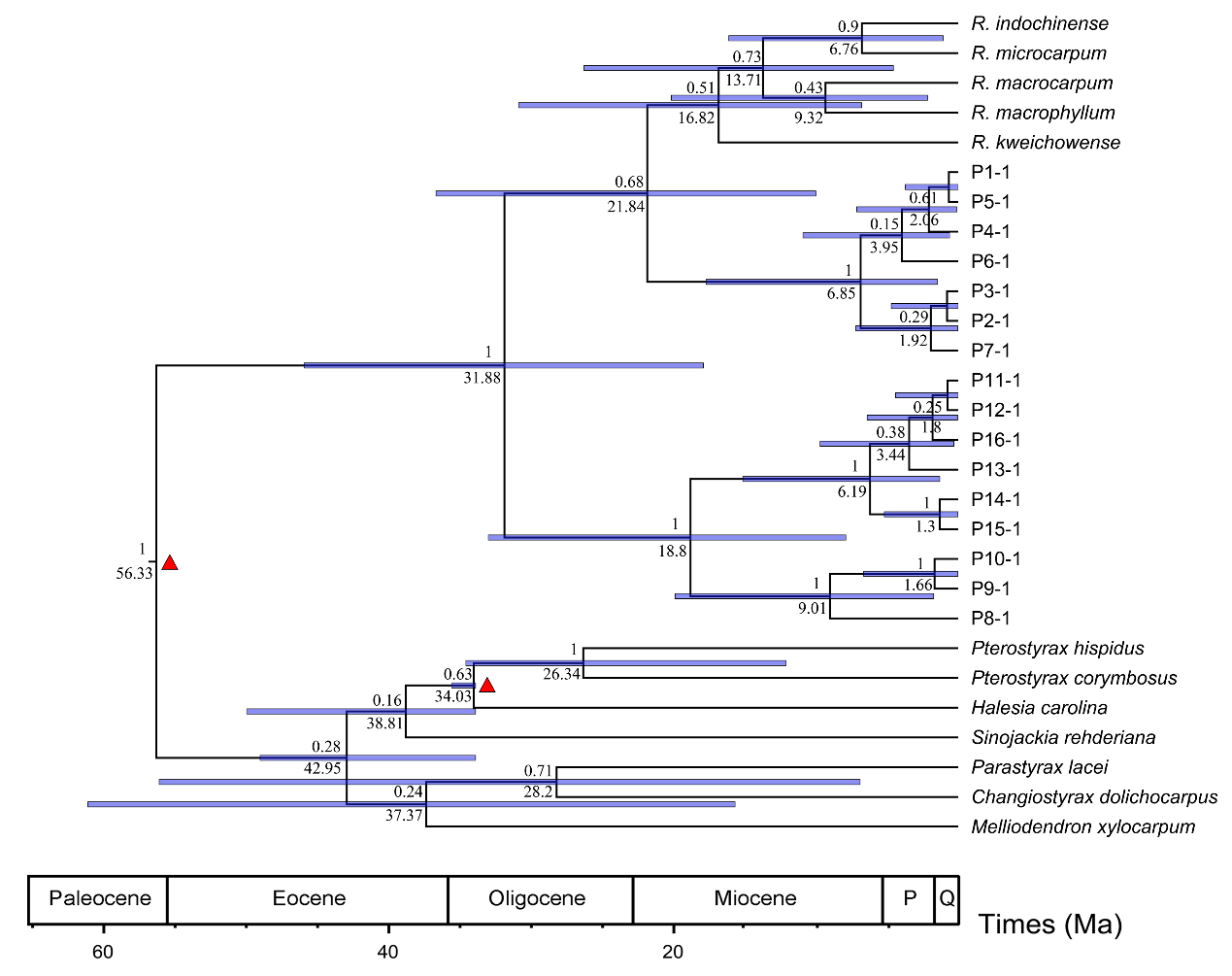


Supplementary Fig. 2 Phylogenetic tree and divergent time estimation of *R. kwangtungense* based on the ribosomal DNA. The fossil points are marked with triangles. Bayesian posterior probabilities (PP) are shown above the bars. The median ages of the nodes are shown below the branches (those younger than 1 Ma are not displayed), with blue bars indicating the 95% highest posterior density intervals.
