# Supplementary figures and images for: Which contributes more to the relict flora distribution pattern in East Asia, geographical processes or climate change? New evidence from the phylogeography of Rehderodendron kwangtungense
Source: BMC Plant Biol. 2024 May 27;24:459. doi: 10.1186/s12870-024-05181-7 (PMC11129394; doi:10.1186/s12870-024-05181-7)

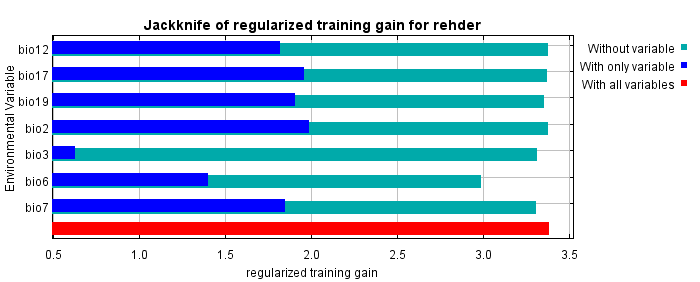


Supplementary Fig. 4 The jackknife test of regularized training gain for *R. kwangtungense.*

Supplement: Supplementary file 5 — Supplementary Material 5 [file 12870_2024_5181_MOESM5_ESM.docx]
